# Mesenchymal Transitions in Development and Disease

**DOI:** 10.1155/2016/5107517

**Published:** 2016-08-02

**Authors:** Damian Medici, Pura Muñoz-Cánoves, Pan-Chyr Yang, Silvia Brunelli

**Affiliations:** ^1^Departments of Orthopaedics and Medicine, The Warren Alpert Medical School of Brown University, Providence, RI 02903, USA; ^2^Department of Experimental and Health Sciences, Pompeu Fabra University (UPF), ICREA and CIBERNED, 08003 Barcelona, Spain; ^3^Centro Nacional de Investigaciones Cardiovasculares (CNIC), 28029 Madrid, Spain; ^4^Department of Internal Medicine and NTU Center of Genomic Medicine College of Medicine, National Taiwan University, Taipei 70101, Taiwan; ^5^Institute of Biomedical Sciences, Academia Sinica, Taipei 11529, Taiwan; ^6^School of Medicine and Surgery, University of Milano-Bicocca, 20900 Monza, Italy

The ability of epithelial cells and endothelial cells to transform into mesenchymal cells is one of the most basic cellular mechanisms in biology. This process, referred to as epithelial-mesenchymal transition (EMT) or endothelial-mesenchymal transition (EndMT), regulates various stages of embryonic development and contributes to the progression of a wide array of diseases and in tissue repair [1, 2].

During embryogenesis, EMT is essential for gastrulation, primitive streak formation, somite dissociation, neural crest development, and palate and lip fusion [3]. EndMT is critical for cardiac development, particularly in the formation of the valves and septa of the heart [4] and the generation of mesodermal cells and multipotent progenitors [5].

In the adult organism, EMT and EndMT are usually dormant until pathological stimuli awaken this embryonic mechanism. For example, EMT is the primary mechanism of cancer metastasis [6, 7], whereas EndMT forms cancer-associated fibroblasts in the tumor microenvironment [8]. Also, both EMT and EndMT have been shown to generate fibroblasts that cause the formation of scar tissue after tissue injury or in association with inflammatory and fibrotic diseases [9–11].

Mesenchymal transitions have traditionally been considered to have a positive effect in development and a negative effect in disease. However, novel findings regarding the stem cell phenotype generated by EMT and EndMT [12, 13] suggest that they may have therapeutic potential for the treatment of various degenerative diseases. This marks an exciting period in this field of research, which may provide new methods for tissue engineering and regeneration by harnessing the power of this embryonic mechanism.

In this special issue, the articles focus on the cutting-edge research on EMT/EndMT, including the role of this mechanism in regenerative medicine, peritoneal fibrosis, liver fibrosis, systemic sclerosis, and angiogenesis. This issue also explores how factors such as mechanical force, vitamin D signaling, and noncoding RNAs regulate mesenchymal transitions, which may provide novel insight into future avenues of research and therapeutic development.

*Damian Medici* *Damian Medici* *Pura Muñoz-Cánoves* *Pura Muñoz-Cánoves* *Pan-Chyr Yang* *Pan-Chyr Yang* *Silvia Brunelli* *Silvia Brunelli*
